# Intestinal Protozoal Parasites in Diarrheal Children and Associated Risk Factors at Yirgalem Hospital, Ethiopia: A Case-Control Study

**DOI:** 10.1155/2014/357126

**Published:** 2014-10-29

**Authors:** Teshome Firdu, Fufa Abunna, Mekonnen Girma

**Affiliations:** ^1^Biology Department, Madawalabu University, P.O. Box 247, Bale Robe, Ethiopia; ^2^Department of Clinical Studies, Addis Ababa University, P.O. Box 34, Bishoftu, Oromia, Ethiopia; ^3^Microbiology-Parasitology Unit, College of Medicine and Health Sciences, Hawassa University, P.O. Box 1560, Hawassa, Ethiopia

## Abstract

*Aim.* A case-control study was conducted to determine the prevalence of *G. lamblia*, *Cryptosporidium*, spp and *E. histolytica/dispar* in diarrheal children at Yirgalem Hospital from February 2011 to August.* Subjects and Methods.* A total of 230 children participated in the study of which 115 (50%) were cases and 115 (50%) were controls. A single stool sample was collected and examined by direct saline wet mount, formol-ether concentration, and modified Ziehl-Neelsen.* Results.* Eighty-four (36.52%) were positive for at least one intestinal parasites (57 (49.56%) from diarrheal children and 27 (23.47%) out of nondiarrheal children). The prevalence of *G. lamblia*, Cryptosporidium spp, and *E. histolytica/dispar* was 15.65%, 9.56%, and 4.35% in children with diarrhea and 1.74%, 5.21%, and 1.74% in those without it, respectively. *Cryptosporidium* spp and *E. histolytica/dispar* revealed higher infection in males (10.81% and 5.4%, resp.) than in females (7.32% and 2.43%, resp.). *G. lamblia* infection was higher in females (29.27%) than in males (8.11%). *Cryptosporidium* spp infection was higher in the age groups of ≤4 years old (53.84%). Significant difference was seen between 10 and 13 (7.69%) years old. Higher prevalence of *E. histolytica/dispar* was found in 5–9 years (85.71%) than ≤4 years old (14.28%). *Conclusion. Cryptosporidium* spp, *E. histolytica/dispar*, and *G. lamblia* were higher in children with diarrhea than in those without it.

## 1. Introduction 

Parasitic protozoa that infects intestinal tract includes* Entamoeba histolytica*,* Giardia lamblia*, and* Cryptosporidium *species, the causative agents of amoebiasis, giardiasis, and cryptosporidiosis, respectively. These organisms are common causes of diarrhea in children. Asymptomatic infection is also common in this population [1].

Like other developing countries, the prevalence of intestinal parasites is widely spread in Ethiopia. Among the common intestinal protozoan parasites* Giardia*,* Cryptosporidium*, and helminthes like* Ascaris* are widely distributed [2]. Reports from different parts of the country showed different prevalence rates of giardiasis and cryptosporidiosis. Study conducted in pediatric diarrheal and nondiarrheal patients in Addis Ababa Hospitals [3] proved infection of* C. parvum* (8.1%) and other parasites like* A. lumbricoides* (0.5%),* G. lamblia* (6.3%), and* E. histolytica/dispar* (1.4%). Similar study conducted in Wondo Genet, southern Ethiopia, also confirmed the prevalence of* G. lamblia* (13.2%) and* E. histolytica/dispar* (0.35%) [4]. In addition, a number of studies and routine diagnosis in Ethiopia pointed out that amoebiasis is one of the most widely dispersed diseases [5]. In a nationwide study of amoebiasis (in 97 communities), the overall prevalence of* E. histolytica* infections as measured by rate of cyst-passers in nonschool communities was 3.5% [6]. Thus, based on this point of view, this study was intended to determine the prevalence of intestinal parasites (*G. lamblia, Cryptosporidium *spp,* and E. histolytica/dispar*) in diarrheal children with insight to the associated risk factors at Yirgalem Hospital, South Ethiopia.

## 2. Materials and Methods

### 2.1. Description of the Study Area and Study Design

**Figure 1:**
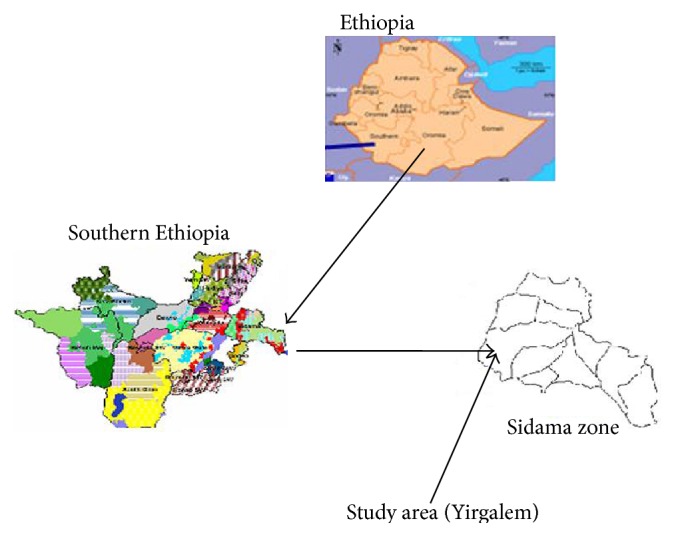
Map of the study area.

The study was carried out at Yirgalem Hospital, located at 317 kilometers south of Addis Ababa, Ethiopia. The area has a latitude and longitude of 6°45′N 38°25′E/6.75°N 38.417°E/6.75; 38.417 and an elevation of 1776 m.a.s.l. (Figure 1).

A case-control study design was used to determine the infection of* E. histolytica/dispar, G. lamblia,* and* Cryptosporidium* spp in diarrheal children and compared with nondiarrheal children. The sample size was determined by considering the prevalence of* Cryptosporidium *spp from pediatric diarrheal patients, which was 8.1% at Addis Ababa by Adamu et al. [3] in Ethiopia. Therefore, a total of 230 children participated in the study of which, 115 were cases and 115 were controls. Children with diarrhea and abdominal problem (discomfort) were considered as cases and children without diarrhea and abdominal discomfort as control during the study period. Diarrhea in this paper is defined a subjective report from study participants/parents/ as having passage of unformed stool for more than 2 or 3 times per day. Children of both sexes of ages under 13 years were included where as children greater than thirteen years old and taking antiparasite treatment were excluded from the study. The study population was chosen consecutively according their arrival during study period.

### 2.2. Parasitological Methods

Single fecal sample from each child was collected with proof and tightly cupped and sterile stool cup. All stool samples were labeled with children's identification number which was given in the sequence of their registration during treatment, and then the results were combined with demographic information of children's such as age and sex. Identification of parasites was based on the morphology of trophozoites, cysts, Oocysts, and ova (diagnostic stages). Each specimen was then examined by direct saline wet-mount, formol-ether concentration and also stained by modified Ziehl-Neelsen(MZN) to detect the Oocysts of* Cryptosporidium* spp [7]. Structured questionnaire was utilized to collect factors possibly causing differences in prevalence of intestinal parasites such as source of drinking water, level of education, the presence of a latrine, and other social and environmental factors.

### 2.3. Statistical Analysis

Statistical analysis was performed by using STATA version 9. An association between the prevalence of the parasites and the identified risk factors was performed using the Chi-square (*χ* ^2^) test. Multivariate logistic regression was also performed for factors obtained significant by *χ* ^2^ test. All values were considered statistically significant at *P* < 0.05.

## 3. Results

In this study, 230 children were participated of which 95 were females and 135 were males. The percentage of males was 58.69% of which 71 (52.59%) were from rural and 64 (47.4%) from urban. Similarly, females account 41.3% of which 48 (50.52%) were from rural and 47 (49.47%) from urban (Table 1).

The prevalence of intestinal parasites in case was 57 (49.56%) and 27 (23.47%) in control group. The majority of the isolates were eggs of* Ascaris lumbricoides* 19 (16.5%) in cases and 11 (9.56%) in controls, followed by trophozoites and cyst stage of* Giardia lamblia* 18 (15.65%) in cases and 4 (3.48%) in controls,* Cryptosporidium *spp Oocyst11 (9.56%) in cases and 2 (1.74%) in controls,* Entamoeba histolytica/dispar* cyst and trophozoites, and ova of other helimenths (Table 2).

The overall prevalence of* G. lamblia*,* Cryptosporidium* spp, and* E. histolytica/dispar* across ages in cases was 31 (26.95%) and 8 (6.95%) in control, which was significantly higher in cases (*P* = 0.0001) (Table 3).

*Giardia lamblia* was more prevalent in the age groups of less than or equal to four years old 10 (45.45%) than five to nine and ten to thirteen which were 8 (36.36%) and 4 (18.18%), respectively. Similarly, the prevalence was not significantly different between age groups of less than or equal to four 10 (45.45%) and ten to thirteen years old 4 (18.18%) (*P* = 0.1027) and no significant difference was observed between five to nine 8 (36.36%) and ten to thirteen 4 (18.18%) years old (*P* = 0.2468).* Cryptosporidium* spp was more prevalent in the age groups of less than or equal to four years old 7 (53.84%) than in five to nine 5 (38.46%) but the difference was not significant (*P* = 0.2196). However, it was significantly different when compared with ten to thirteen years old 1 (7.69%) (*P* = 0.0088) and no significance difference was seen in the age of five to nine 5 (38.46%) and ten to thirteen 1 (7.69%) years old (*P* = 0.067). Furthermore, the prevalence of* E. histolytica/dispar* was more in the age group of five to nine 6 (85.71%) than in the age groups of less than or equal to four years old 1 (14.28%) with significance difference (*P* = 0.0355). The total prevalence of* G. lamblia*,* Cryptosporidium* spp, and* E. histolytica/dispar* was compared among the three age groups. A significant difference was not seen between age groups of less than or equal to four 18 (46.15%) and five to nine 19 (48.71%) (*P* = 0.8704), but significant between ten to thirteen years old 5 (12.82%) (*P* = 0.0046) (Table 4).

Having animal contact and disposing waste in compound were significant risk factors (*P* < 0.05) for* G. lamblia* positive children. As well, no breast fed, child education/schooling, and no hand washing after toilet were significant risk factors for* Cryptosporidium* spp positive children (*P* < 0.05). Moreover, improper storage of food and drinks and contact with animal were the identified risk factors for* E. histolytica/dispar *positive children (*P* < 0.05). The associated risk factors are given with noted *P* values by Chi-square (*χ* ^2^) test as below (Table 5).

Values obtained significant in *χ* ^2^ test were subjected to multivariate logistic regression for further analysis. From multivariate analysis disposing waste in the compound was a significant risk factor for* Cryptosporidium* spp positive children (*P* = 0.016). Correspondingly, no proper food storage was a significant risk factor for* E. histolytica/dispar* positive children (*P* = 0.029) (Table 6).

## 4. Discussion

The overall prevalence of intestinal parasites in this study is 84 (36.52%) which was higher than the one reported from Quetta Hospital, 31% by Ahsan-ul-Wadood et al. [8] in Pakistan and lower than the one reported by Fatemeh et al. [9] in Iran, 47.7%. In this study, infection with* G. lamblia* in stool samples from children is 15.65%. The finding was different with studies conducted in Ethiopia and other countries. A study in Gondar teaching hospital by Huruy et al. [10] revealed 5% which is lower from this study. In addition, study from Pakistan by Adnan et al. [11] revealed 10.3% and Ejiofor et al. [12] from South East Nigeria showed 10.1% and Da'as [13] reported 4.4% from Palestine. This difference in this study might be due to the hygienic practice or environmental factors and the society's awareness toward this parasite and or educational status of children's family or better living conditions of children among these countries. On the other hand, the finding is also different from the one reported from Ethiopia, Addis Ababa by Adamu et al. [3] from pediatric diarrheal children which was 6.3% and Liza et al. [4] from south Ethiopia, Wondo Genet town which was 13.2%. This indicates that this parasite is prevalent in Yirgalem area. The difference from Liza et al. [4] south Ethiopia, Wondo Genet town, might be due to the study participant variation, where no diarrhea was seen in these children.

On the basis of age, the finding is in agreement with Ejiofor et al. [12] study from South east Nigeria in Awka, where high prevalence was reported in age groups of six months to four years old (55.6%) and lowers between age group of ten to thirteen years old (11.1%). However, it is inconsistent with the study in Guma from Nigeria by Nyamngee et al. [14] where higher prevalence was in the age group of five to nine years old (48.3%) than in the age of less than or equal to four years old (18.7%) and ten to thirteen years old (33%). The reason for this age group (less than or equal to four years old) vulnerability in this study might be explained by milk bottles contamination or unbreast feeders and creeping on a contaminated grounds and accessing dirty material (especially fecally contaminated water and soil) into their mouth Adnan et al. [11]. In addition, this age group children use diaper which may allow the transmission via hand to mouth contamination if not used properly.

Sex-based result of this study discloses high prevalence of* G. lamblia* in females (29.27%) than in males (8.11%) significantly (*P* = 0.0034). This is not supported with Al-Saeed and Issa [15] in Dohuk from Iraq, where high prevalence was reported in males (41.6%) and low in females (35.6%) and Nyamngee et al. [14] in Guma from Nigeria reported high prevalence in males (56.5%) than in females (43.5%). The possible reason for this finding is that females' practical activity in Ethiopia like fetching water for their family and some indoor activity. The associated risk factors were analyzed in the current study to look at the possible source of infection; however, no significant factors were seen (*P* < 0.05). The prevalence of* G. lamblia* among different water sources (river, pipe, and spring) of the study participant reveals high infection in river water users. However, the prevalence was not significantly different in river water users (*P* > 0.05). This may show that the entire water source in the study area might be the source of infection. Because the cyst of the parasite is not be killed by common water disinfectants or the infection is not only restricted to water sources used.

The result of this study demonstrates that the prevalence of* Cryptosporidium* spp is 9.56% among diarrheal children at Yirgalem Hospital. The figure is different more or less relative to the prevalence of this parasite reported in other studies conducted in several parts of Ethiopia and other countries. Adamu et al. [3] studied the prevalence of intestinal parasites in pediatric diarrheal children in Addis Ababa and showed 8.1% of* Cryptosporidium parvum *in stool of children aged less than 5 years. However, in this study,* Cryptosporidium* spp infection is (9.56%) which is higher. This could be due to the fact that the study participants in this study were under 13 and also in this study all* Cryptosporidium* species were considered as* Cryptosporidium* spp. Furthermore, variation might be arisen from the variation of hygienic practice performed between the two environments. Nevertheless, the study is in agreement with reported prevalence of* C. parvum* from developing countries which was 4–32% [16]. The result showed that 14.4% of children greater than 5 years old had* Cryptosporidium* spp in their stool samples. The present study is in agreement with the finding of Mumtaz et al. [17] which was 9% from pediatric unit of North West Pakistan and Jacobsen et al. [18] 8.9% in young children in Ecuador. In this study,* Cryptosporidium* Oocyst is more frequently detected in children less than or equal to 4 years old (53.84%), followed by five to nine years of old (38.46%) and ten to thirteen years of old (7.69%) with no significant difference (*P* > 0.05). This age based prevalence of* Cryptosporidium* spp is in line with Mumtaz et al. [17] from Pakistan, Adamu et al. [3] in Ethiopia and Da'as [13] in Palestine.* Cryptosporidium* spp infection in relation to sex in this study reveals that males are more susceptible to infection (10.81%) than females (7.32%), insignificantly (*P* = 0.5434). However, this result is in agreement with Mumtaz et al. [17], who reported higher prevalence of infection among males (72.2%) as compared to female (27.8%) children. Males being more susceptible to this infection might be attributed to the genetic variability which may require further investigation. Of the analyzed risk factors, no hand washing after toilet was seen the significant risk factor (*P* < 0.05). This is in agreement with Molbak et al. [19] from Guinea Bissau and Mumtaz et al. [17] in Pakistan. Patients using river, pipe, and spring water for domestic purpose were found to be infected with this infection, 54.54%, 27.3%, and 18.2% of infection, respectively. Infection was higher among those who use river water. However, the difference among water sources and* Cryptosporidium* spp infection was not seen significant statistically (*P* = 0.295), which is in agreement with Da'as [13]. The possible reason could be due to the ability of the Oocysts to survive the common sterilizing agents of water. In addition, the possible explanation for the absence of variation among the water source in this study might be attributed to whatever water source used, the infection was not restricted to water source.

In this study, the prevalence of* E. histolytica/dispar* is low (4.35%) relative to both* Cryptosporidium* spp (9.56%) and* G. lamblia *(15.65%) across all age groups and sex of the study participants. This finding is higher than the findings in Damghan from Iran by Heidari and Rokni [20], which was 2.3%. This variation might be due to the differences in the study population in which they were selected from day care centers and in this case the majority of the subjects could be healthy unless some asymptomatic cases might be presented. However, in this study, the study populations were children of pediatrics patients. On the other hand the difference might be due to families' awareness toward parasitic infection and the way of handling their child, economic status, and climatic condition. Furthermore, it is not in line with Dawah et al. [21], in Kaduna Metropolis where 14.3% of* E. histolytica/dispar* was reported. The difference might be sourced from the technique employed, because microscope together with ELISA technique was employed in the study whereas, this study was based on microscope only. However, this study coincides with Al-Harthi and Jamjoom [22] from Makkah where the finding was 4.3%. Furthermore, the result is different from studies conducted in different parts of Ethiopia. A study conducted by Adamu et al. [3] in Addis Ababa revealed 1.4% prevalence and Liza et al. [4] at Wondo Genet showed 0.35% both of which were lower than this finding. But this finding is almost in conformity with Kloos and Tesfayohanis [5] study, which revealed 3.5% from nationwide study of amebiasis in 97 communities and the overall prevalence of* E. histolytica* infections, as measured by rate of cyst-passers in nonschool communities.

The relationship between sex and* E. histolytica/dispar* in children was assessed in this study. According to the result, the infection of* E. histolytica/dispar* is more prevalent in males (5.4%) as compared to females (2.43%), but no significant difference was seen (*P* = 0.4557). The finding on the basis of sex in this study is not supported with the finding of Munazza et al. [23] from Pakistan where high prevalence was recorded in females (31.5%) than in males (19.6%). However, it is in agreement with Chabalala and Mamo [24], in Nakuru district where higher prevalence was reported in males than in females from Kenya. Males are more susceptible than females to infections caused by parasites; males generally exhibit reduced immune responses and increased intensity of infection compared to females [25, 26]. These differences are usually attributed to ecological (sociological in humans); and physiological, usually hormonal in origin. Ecological factors include differential exposure to pathogens because of sex-specific behavior or morphology [27]. Other proximate cause of sex differences in infection is differences in endocrine-immune interactions [25]. Sex steroid hormones also alter genes and behaviors that influence susceptibility and resistance to infection. Thus, males may be more susceptible to infection than females not only because androgens reduce immunocompetence, but because sex steroid hormones affect disease resistance genes and behaviors that make males more susceptible to infection [25].

The relationship between age and the prevalence of* E. histolytica/dispar* reveals that higher prevalence is recorded among age groups of five to nine (85.71%) than less than or equal to four years old (14.28%) with significant difference (*P* = 0.0355). The finding is in line with Caballero-Salcedo et al. [28] study in Mexico where 11% of the tested population aged five to nine years old was infected with amoeba. However, this finding is not supported with Munazza et al. [23], from Pakistan where finding in the prevalence of* E. histolytica/dispar* was higher among age groups of one to five years old. But this study is in line with Astal [29], who reported prevalence of* E. histolytica/dispar* from Khan Younis, Government Hospital with the prevalence of 34.2% in age group of six to eleven years old children than other age group in Palestine. The prevalence decreases with age increment in the present study. Children in this age group are free to play anywhere irrespective of the cleanliness or dustiness area while the younger ones are quite and protected by their parents. Playing areas are the main sources of infection, because waste materials of homes might be thrown there, which might be the source of* E. histolytica/dispar* infection. Additionally children in these age groups are independent in use of toilet and other activities. This study shows improper food storage as the associated risk factor for* E. histolytica/dispar* (*P* < 0.05). Having no proper food storage might be favorable for insects/house flies to spread this parasite Adnan et al. [11].

## 5. Conclusion

The prevalence of* Cryptosporidium *spp,* E. histolytica/dispar*, and* G. lamblia* in children with diarrhea is higher than in those without it. The prevalence* Ascaris lumbricoides* was higher both in case and controls. The occurrence of the parasites is associated with disposing waste in compound, improper food, and drink storage. Therefore, constant appropriate health education for community and molecular typing of these protozoan isolates should be carried out.

## Figures and Tables

**Table 1:** Sociodemographic distribution of cases and controls in a study of intestinal parasites infections in diarrheal children.

Category	Cases (*N* = 115) no (%)	Controls (*N* = 115) no (%)	Total no (%)
Age (in year)			
≤4	51 (44.34)	33 (28.69)	84 (36.52)
5–9	43 (37.39)	49 (42.6)	92 (40)
10–13	21 (18.26)	33 (28.69)	54 (23.48)
Sex			
Male	74 (64.35)	61 (53)	135 (58.69)
Female	41 (35.65)	54 (47)	95 (41.3)
Residence			
Rural	64 (55.65)	55 (47.82)	119 (52.17)
Urban	51 (44.34)	60 (52.17)	111 (48.26)

**Table 2:** The overall prevalence of intestinal parasites in diarrheal (cases) and nondiarrheal (controls) children.

Parasites	Children with diarrhea and GI problem *n* (%)	Children without diarrhea and GI problem *n* (%)	*P* values
Protozoan			
*G*.* lamblia *	18 (15.65)	4 (3.48)	0.0019^*^
*Cryptosporidium *spp	11 (9.56)	2 (1.74)	0.0109^*^
*E*.* histolytica/dispar *	5 (4.35)	2 (1.74)	0.2506
Helimenths			
*A. lumbricoides *	19 (16.5)	11 (9.56)	0.1194
*H. nana *	2 (1.74)	1 (0.87)	0.5616
*H. worm *	5 (4.35)	3 (2.61)	0.4723
*T. trichuria *	4 (3.48)	4 (3.48)	1.000
*S. stercoralis *	1 (0.87)	0 (0)	0.3172
Total	**57 (49.56)**	**27 (23.47)**	0.0000^*^

^*^Significance difference at *P* < 0.05.

GI: Gastro intestinal.

**Table 3:** Prevalence of* G*.* lamblia*, *Cryptosporidium* spp, and *E. histolytica*/*dispar* in case and control groups across age.

Parasites	Cases (*N* = 115)				Controls (*N* = 115)			
	Age (in year)				Age (in year)			
	≤4	5–9	10–13	Total	≤4	5–9	10–13	Total
	*n* (%)	*n* (%)	*n* (%)	*n* (%)	*n* (%)	*n* (%)	*n* (%)	*n* (%)

*G. lamblia *	9 (50)	6 (33.33)	3 (16.67)	18 (15.65)	1 (25)	2 (50)	1 (25)	4 (3.47)
*Cryptosporidium *spp	7 (63.63)	4 (36.36)	0	11 (9.56)	0	1 (50)	1 (50)	2 (1.74)
*E. hist/dispar *	1 (20)	4 (80)	0	5 (4.35)	0	2 (100)	0	2 (1.74)
Total	**17 (54.83)**	**14 (45.16)**	**3 (9.68)**	**31 **(**26.95**)^*^	**1 (14.28)**	**5 (62.5)**	**2 (25)**	**8 **(**6.95**)^*^

^*^Significance difference at *P* < 0.05.

**Table 4:** The combined prevalence of *G. lamblia*, *Cryptosporidium* spp, and* E. histolytica/dispar* across age.

Parasites	(*N* = 230)			
	Age groups (in year)			
	≤4 no (%)	5–9 no (%)	10–13 no (%)	Total no (%)
*G. lamblia *	10 (45.45)	8 (36.36)	4 (18.18)	22 (9.56)
*Cryptosporidium *spp	7 (53.84)	5 (38.46)	1 (7.69)	13 (5.65)
*E. histolytica/dispar *	1 (14.28)	6 (85.71)	0 (0)	7 (3.04)
Total	**18 (46.15)**	**19 (48.71)**	**5 (12.82)**	**39 (16.95)**

**Table 5:** Associated risk factors for *G. lamblia*, *Cryptosporidium* spp, and* E. histolytica/dispar *positives in cases.

Risk factors	Option	*G. lamblia *	P values	*Cryptosporidium *spp	*P* values	*E. histolytica/dispar *	*P* values

Residence	Rural	10 (55.56)	0.928	7 (63.6)	0.254	3 (60)	0.873
	Urban	8 (44.44)		4 (36.4)		2 (40)	

Mothers educ.	Educated	8 (44.44)	0.297	4 (36.4)	0.176	2 (40)	0.471
	Illiterate	10 (55.56)		7 (63.6)		3 (60)	

Breast fed	Yes	3 (16.67)	0.512	5 (45.45)	0.008^*^	0 (0)	0.217
	No	15 (83.33)		6 (54.54)		5 (100)	

Childs educ.	Schooling	9 (50)	0.380	4 (36.4)	0.002^*^	4 (80)	0.231
	None	9 (50)		7 (63.6)		1 (20)	

Animal contact	Yes	14 (77.78)	0.027^*^	9 (81.8)	0.051	5 (100)	0.035^*^
	No	4 (22.22)		2 (18.2)		0	

CDc	Yes	15 (88.23)	0.079	5 (45.5)	0.542	5 (100)	0.089
	No	3 (16.67)		3 (27.3)		0	

PFS	Yes	9 (50)	0.196	4 (36.4)	0.050	2 (40)	0.003^*^
	No	9 (50)		7 (63.6)		3 (60)	

Water source	Pipe	7 (38.89)	0.203	3 (27.3)	0.295	2 (40)	0.381
	River	9 (50)		6 (54.54)		3 (60)	
	Spring	2 (11.11)		2 (18.2)		0	

DWIC	Yes	6 (33.33)	0.007^*^	6 (54.5)	0.892	5 (100)	0.902
	No	12 (66.67)		5 (45.5)		0	

HWAT	Yes	8 (44.44)	0.055	4 (36.4)	0.031^*^	2 (40)	0.226
	No	10 (55.56)		7 (63.6)		3 (60)	

HWWF	No	11 (16.42)	0.100	5 (45.5)	0.156	3 (60)	0.255
	Yes	7 (38.89)		6 (54.5)		2 (40)	

^*^Significance at *P* < 0.05.

PFS: proper food storage, DWIC: disposing waste in compound, and HWAT: hand washing after toilet.

HWWF: hand washing when feeding, CDc: cow dung contact. Educated: those who have learned at some school level and have knowledge concerning parasites infection.

**Table 6:** Multivariate logistic regression of significant risk factors from Chi-square (*χ* ^2^) test.

Risk factors	Options	*G. lamblia *	*P* values	*Cryptosporidium* spp	*P* values	*E. histolytica/dispar *	*P* values
Breast fed	Yes	3 (16.67)	0.312	5 (45.45)	0.099	0 (0)	0.896
	No	15 (83.33)		6 (54.54)		5 (100)	

Childs educ.	Schooling	9 (50)	0.271	4 (36.4)	0.120	4 (80)	0.184
	None	9 (50)		7 (63.6)		1 (20)	

Animal contact	Yes	14 (77.78)	0.422	9 (81.8)	0.077	5 (100)	0.874
	No	4 (22.22)		2 (18.2)		0	

PFS	Yes	9 (50)	0.902	4 (36.4)	0.198	2 (40)	0.029^*^
	No	9 (50)		7 (63.6)		3 (60)	

DWIC	Yes	6 (33.33)	0.053	6 (54.5)	0.149	5 (100)	0.270
	No	12 (66.67)		5 (45.5)		0	

HWAT	Yes	8 (44.44)	0.800	4 (36.4)	0.016^*^	2 (40)	0.754
	No	10 (55.56)		7 (63.6)		3 (60)	

^*^Significance difference at *P* < 0.05.

PFS: Proper food storage, HWWF: hand washing when feeding, DWIC: disposing waste in compound, CDc: cow dung contact, and HWAT: hand washing after toilet.
